# The Chief Scientist Office Cardiovascular and Pulmonary Imaging in SARS Coronavirus disease-19 (CISCO-19) study

**DOI:** 10.1093/cvr/cvaa209

**Published:** 2020-07-23

**Authors:** Kenneth Mangion, Andrew Morrow, Catherine Bagot, Hannah Bayes, Kevin G Blyth, Colin Church, David Corcoran, Christian Delles, Lynsey Gillespie, Douglas Grieve, Antonia Ho, Sharon Kean, Ninian N Lang, Vera Lennie, David J Lowe, Peter Kellman, Peter W Macfarlane, Alex McConnachie, Giles Roditi, Robert Sykes, Rhian M Touyz, Naveed Sattar, Ryan Wereski, Sylvia Wright, Colin Berry

**Affiliations:** c1 British Heart Foundation Glasgow Cardiovascular Research Centre, Institute of Cardiovascular and Medical Sciences, University of Glasgow, Glasgow, UK; c2 Department of Cardiology, Queen Elizabeth University Hospital, NHS Greater Glasgow and Clyde Health Board, Glasgow, UK; c3 Department of Haemostasis and Thrombosis, Royal Infirmary, NHS Greater Glasgow and Clyde Health Board, Glasgow, UK; c4 Department of Respiratory Medicine, Glasgow Royal Infirmary, NHS Greater Glasgow and Clyde Health Board, Glasgow, UK; c5 Department of Respiratory Medicine, Queen Elizabeth University Hospital, NHS Greater Glasgow and Clyde Health Board, Glasgow, UK; c6 Institute of Cancer Sciences, University of Glasgow, Glasgow, UK; c7 Project Management Unit, Glasgow Clinical Research Facility, Greater Glasgow and Clyde Health Board, Glasgow, UK; c8 Department of Respiratory Medicine, Royal Alexandra Hospital, NHS Greater Glasgow and Clyde Health Board, Glasgow, UK; c9 MRC-University of Glasgow Centre for Virus Research, Glasgow, UK; c10 Robertson Centre for Biostatistics, Institute of Health and Wellbeing, University of Glasgow, Glasgow, UK; c11 Department of Cardiology, University Hospital Ayr, Ayrshire and Arran Health Board, Ayr, UK; c12 Department of Emergency Medicine, Queen Elizabeth University Hospital, NHS Greater Glasgow and Clyde Health Board, Glasgow, UK; c13 National Heart, Lung, and Blood Institute, National Institutes of Health, DHHS, Bethesda, MD, USA; c14 Electrocardiography Core Laboratory, Institute of Health and Wellbeing, University of Glasgow, UK; c15 Department of Radiology, NHS Greater Glasgow and Clyde Health Board, Glasgow, UK; c16 Department of Cardiology, Royal Infirmary, NHS Greater Glasgow and Clyde Health Board, Glasgow, UK; c17 British Heart Foundation Centre for Cardiovascular Science, University of Edinburgh, Edinburgh, UK; c18 Department of Pathology, Laboratory Medicine, Queen Elizabeth University Hospital, NHS Greater Glasgow and Clyde Health Board, Glasgow, UK

**Keywords:** SARS-CoV-2, Myocardial inflammation, Myocardial infarction, Myocardial injury, Imaging, Biomarkers

## Abstract

**Background:**

COVID-19 is typically a primary respiratory illness with multisystem involvement. The prevalence and clinical significance of cardiovascular and multisystem involvement in COVID-19 remain unclear.

**Methods:**

This is a prospective, observational, multicentre, longitudinal, cohort study with minimal selection criteria and a near-consecutive approach to screening. Patients who have received hospital care for COVID-19 will be enrolled within 28 days of discharge. Myocardial injury will be diagnosed according to the peak troponin I in relation to the upper reference limit (URL, 99th centile) (Abbott Architect troponin I assay; sex-specific URL, male: >34 ng/L; female: >16 ng/L). Multisystem, multimodality imaging will be undertaken during the convalescent phase at 28 days post-discharge (Visit 2). Imaging of the heart, lung, and kidneys will include multiparametric, stress perfusion, cardiovascular magnetic resonance imaging, and computed tomography coronary angiography. Health and well-being will be assessed in the longer term. The primary outcome is the proportion of patients with a diagnosis of myocardial inflammation.

**Conclusion:**

CISCO-19 will provide detailed insights into cardiovascular and multisystem involvement of COVID-19. Our study will inform the rationale and design of novel therapeutic and management strategies for affected patients.

**Clinical trial registration:**

ClinicalTrials.gov identifier NCT04403607.

## Background

**Figure 1:**
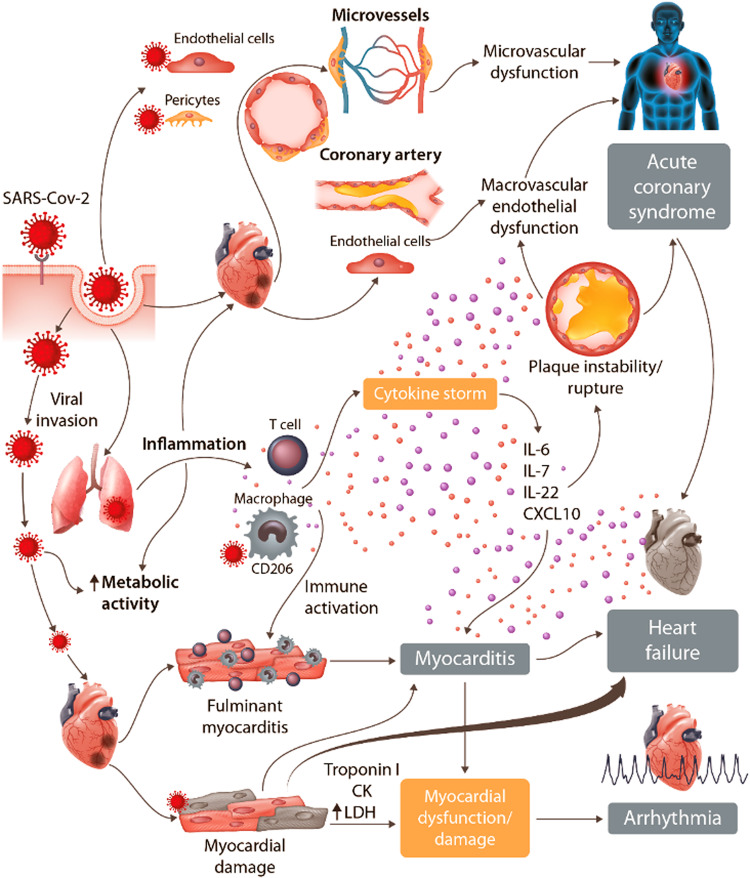
Cardiovascular pathophysiology of COVID-19. With permission of T. Guzik *et al. Cardiovascular Research* 2020.

COVID-19 presents the most significant threat to human health in modern times.^1–4^ The severe acute respiratory syndrome coronavirus 2 (SARS-CoV-2) infection, which causes COVID-19 illness, is mediated by tropism for nasopharyngeal and pulmonary epithelial cells. Cardiovascular complications are common, affecting ∼1 in 4 patients, are associated with prior cardiovascular disease, and increase the risk of death.^5–11^. Cardiovascular injury may be secondary to inflammation, hypoxia, hypotension, and thrombosis, or, potentially, through virus invasion of endothelial cells, vascular smooth muscle cells, and pericytes, leading to vascular injury or dysfunction. This mechanism may then play a primary role in the development of pathology.^11^ The angiotensin-converting enzyme 2 (ACE2) transmembrane receptor, which normally has protective effects in the cardiovascular system, is also the receptor which mediates virus transmission in human cells, including in the cardiovascular system (*Figure 1*).^12^^,^^13^

### Mechanisms of SARS-CoV-2 tropism for cardiovascular cells

Coronavirus (Coronaviridae family) is named after the crown morphology of its outer membrane. SARS-CoV-1, which caused an epidemic in 2002–2003, and SARS-CoV-2, infect human cells when the surface spike (S) protein, a type 1 membrane glycoprotein,^10^^,^^12^ binds to the ACE2 transmembrane receptor protein on human cells. SARS-CoV-1 and SARS-CoV-2 have 76% similarity in their S proteins. Fusion of the coronavirus S protein with the cell surface ACE2 transmembrane receptor internalizes the virus, promulgating replication and dissemination. ACE2 is expressed in endothelial cells in the heart, lungs, kidneys, intestine, and testis,^14–16^ and is a member of the counter-regulatory axis of the renin–angiotensin–aldosterone system (RAAS).^17^ In its canonical function, as a carboxypeptidase, ACE2 is protective in the cardiovascular system through its role in metabolising angiotensin I (Ang I) to Ang-(1-9) and Ang II to Ang-(1-7), therefore reducing Ang II levels and generating the protective peptides Ang-(1-9) and Ang-(1-7) which act through the angiotensin type 2 receptor (AT2R)^18^^,^^19^ and the Mas receptor,^20^ respectively.

### Prior knowledge from SARS-CoV and cardiovascular involvement

Like SARS-CoV-2, SARS-CoV-1 also uses ACE2 and transmembrane protease serine 2 (TMPRSS2) as mechanisms of cell invasion. During SARS-CoV-1 infection of transgenic mice expressing human ACE2, ACE2 becomes dysregulated and depleted.^21^ In acute lung disease triggered by sepsis, ACE2 is directly protective.^22^ SARS patients may experience overwhelming immune and inflammatory responses, in the form of a cytokine storm, leading to left ventricular (LV) systolic dysfunction, arrhythmias, and sudden death.^23^^,^^24^ Imaging is therefore well placed to investigate the underlying pathology of myocardial injury in COVID-19 patients.

### Evidence of coronavirus inoculation of cardiovascular cells and myocarditis

Chan *et al*.^25^ provided preliminary evidence of coronavirus infection in pulmonary endothelial cells. Varga *et al*. studied the histopathology of heart, lung, kidney, and liver tissue samples from three patients who died from COVID-19. They found evidence of viral elements within endothelial cells and endotheliitis.^11^ Recent studies suggest that systemic manifestions of COVID-19, including hypertension, thrombosis, myocardial involvement, and kidney failure, may be due to endothelial and vascular disease.^26^

High levels of ACE2 are expressed by pericytes in the heart^16^ and cardiomyocytes (7.5%, scRNA-seq data).^27^ ACE2 may have paradoxical roles since ordinarily it regulates a vasculoprotective signalling pathway and has protective catalytic effects in the lung. On the other hand, it is a receptor for virus transmission into human cells. SARS-CoV-2 infection reduces the activity and/or protein levels of ACE2, leading to a harmful imbalance of Ang II/AT1R effect.^28^ ACE2 overexpression enhances the stability of atherosclerotic plaque.^29^ However, ACE2 transcript and protein levels are increased in patients with cardiovascular disease such as heart failure,^16^ and post-myocardial infarction (MI),^30^ implying an increased risk of cardiac infection. The association between COVID-19 and RAAS inhibitor therapy^31^^,^^32^ and other cardiovascular medications^21^ is most probably explained by underlying cardiovascular disease.^31^ There have been a number of case reports or case series of reported COVID-19-associated myocardial injury, presenting as myocarditis^2^^,^^5^^,^^33^^,^^34^ or reverse Takotsubo.^35^^,^^36^ A number of autopsy reports identify SARS-CoV-2 RNA in the myocardium of patients dying from COVID-19-related complications (pulmonary embolism and pneumonitis^37^, and undefined^38^), whilst other series do not identify myocardial SARS-CoV-2 RNA in the context of death related to pneumonitis and acute respiratory distress syndrome (ARDS).^39–42^ Taken together, this suggests that there are several potential mechanisms of myocardial injury (endotypes) in severe SARS-CoV-2 infection and that further research is warranted.

### Evidence of venous and pulmonary arterial thrombosis

Endothelial damage and thrombotic microvascular angiopathy may underpin systemic vascular dysfunction.^10^ Autopsy studies have shown evidence of this at a pulmonary microvascular level, and this may be responsible in part for the severe hypoxia present in these patients.^43^

### Evidence of renal involvement

The prevalence of renal involvement in patients with SARS-CoV-2 infection is low,^44^^,^^45^ and is hypothesized to be secondary to cytokine damage, systemic effects of the illness,^44^ or interaction between the cardio-pulmonary axis and renal function, as has been reported in ARDS.^46^

## Contemporary definitions of acute myocardial infarction or myocardial injury

Acute myocardial injury is defined as a rise in the circulating concentration of troponin above the 99th percentile of the upper reference limit and then a fall. Acute MI is myocardial injury in the context of myocardial ischaemia. Acute MI is categorized into one of five types.^47^ Type 1 MI is diagnosed based on the occurrence of at least one of the following: (i) symptoms of acute myocardial ischaemia; (ii) new ischaemic ECG changes; (iii) development of pathological Q waves; (iv) imaging evidence of new loss of viable myocardium or new regional wall motion abnormality in a pattern consistent with an ischaemic aetiology; or (v) identification of a coronary thrombus by angiography including intracoronary imaging or by autopsy. Clinical scenarios of Type 1 MI include coronary plaque rupture and plaque erosion. A diagnosis of Type 2 MI is based on evidence of an imbalance between myocardial oxygen supply and demand unrelated to coronary thrombosis, requiring at least one of the following: (i) symptoms of acute myocardial ischaemia; (ii) new ischaemic ECG changes; (iii) development of pathological Q waves; or (iv) imaging evidence of new loss of viable myocardium or new regional wall motion abnormality in a pattern consistent with an ischaemic aetiology.

Clinical scenarios of Type 2 MI include severe hypertension, hypoxia, and tachyarrhythmia. Type 3 MI is the classification used for patients who suffer cardiac death due to MI, and Types 4 and 5 MI are iatrogenic, consequent on MI arising from percutaneous coronary intervention (PCI) or coronary artery bypass graft (CABG), respectively.^47^ The diagnosis of myocardial infarction with no obstructive coronary arteries (MINOCA) indicates that there is an ischaemic mechanism responsible for the myocyte injury. Clinical scenarios for MINOCA include MI due to atherosclerotic plaque rupture, i.e. Type 1 MI, coronary spasm, spontaneous coronary dissection, and Type 2 MI.

Myocardial injury is diagnosed based on elevated cardiac biomarkers without myocardial ischaemia. Acute myocardial injury is associated with a rise and fall of troponin, whereas chronic myocardial injury is associated with a stable troponin concentration. Myocardial injury may be primarily due to a cardiac or non-cardiac cause.^48^ Cardiac causes of myocardial injury include arrhythmias and disorders of coronary vascular function. Non-cardiac causes of myocardial injury include anaemia and pulmonary embolism.^48^

The plasma concentration of troponin I will be measured using the Abbott Architect assay. The sex-specific upper reference limits (99th centile) are >16 ng/L and >34 ng/L for females and males, respectively.

## Rationale for multimodality imaging

### MRI

Cardiovascular magnetic resonance imaging (CMR) is a non-invasive diagnostic test for myocardial pathology.^49–51^ Through tissue characterization, CMR can differentiate between myocardial inflammation (acute vs. chronic), myocardial infarction (acute vs. chronic scar), and pericarditis, and simultaneously provide information on cardiac function, blood flow, and incidental findings such as pericardial effusion.^49^^,^^50^^,^^52^^,^^53^

In COVID-19, myocardial inflammation due to viral myocarditis, ischaemia, or stress cardiomyopathy, as well as MINOCA (Type 1 or Type 2 MI) and other cardiac complications are potentially more common than previously thought based on current crude biomarker studies. Establishing their real frequency using powerful imaging techniques is essential for optimizing risk stratification and therapy. In our study, MRI will be used to assess for and classify clinical endotypes. The endotypes of myocardial involvement are (1) myocardial inflammation, e.g. (1.1) myocarditis, (1.2) ischaemia, or (1.3) stress (Takotsubo) cardiomyopathy; (2) myocardial infarction; (3) indeterminate; or (4) none. A diagnosis of viral myocarditis typically requires endomyocardial biopsy (EMB). We do not anticipate that EMB will be undertaken in our study population; therefore, a presumptive diagnosis may be made based on the available clinical information.

The wide field of view of the chest and abdomen also permits imaging of the lungs and kidneys.

### CT

A computed tomography (CT) coronary angiogram/chest protocol will image for coronary artery disease (CAD), delayed enhancement, lung perfusion, pulmonary thrombo-embolism, and parenchymal pathology.

Taken together, integration of multimodality imaging of the heart, lungs, and kidneys within a single visit represents a highly novel approach to investigate the cardiovascular complications of COVID-19. The imaging scans will also support advanced computational modelling to better understand this multisystem disease.

## Hypothesis

We hypothesize that myocarditis (myocardial inflammation) is common after SARS-CoV-2 infection.

## Objectives

- To assess the incidence, nature, time-course and clinical significance of cardiovascular involvement in patients with COVID-19.
- To quantify myocardial perfusion as a measure of coronary microvascular function.
- To determine whether patients with COVID-19 and pre-existing cardiovascular disease are at increased risk of cardiac involvement.
- To assess the incidence of pulmonary thrombo-embolism and right ventricular dysfunction.
- To assess for renal involvement in patients with COVID-19 and suspected cardiac involvement
- To assess mechanisms using circulating biomarkers of cardiac injury [troponin I, N-terminal probrain natriuretic peptide (NT-proBNP)], inflammation [C-reactive protein (CRP)], vascular injury [sACE2, von Willebrand factor (vWF), and interleukin-6 (IL-6)], and thrombotic microangiopathy, and their changes over time.
- To establish substudies using computational cardiovascular modelling, electrocardiography, and pathology.

## Study design

The CISCO-19 study has a prospective, observational, multicentre, longitudinal, cohort design, minimal selection criteria, and a near-consecutive approach to screening.

### Study population

The study population will be focused on COVID-19 patients who have survived the initial, acute illness. Accordingly, the findings from our study are most relevant to patients in the convalescent phase looking forward to the longer term. We will characterize cardiac involvement evidenced by an increase in circulating high sensitivity troponin I > upper reference limit at any time during the hospital episode of care. Control data may be drawn from local cohorts, as appropriate. Patients who received hospital care with COVID-19 will be enrolled within 28 days of discharge. This enrolment strategy is intended to maximize enrolment of all-comers affected by COVID-19. Patients who die or are ineligible for other reasons will be recorded in a screening log.

**Figure 2:**
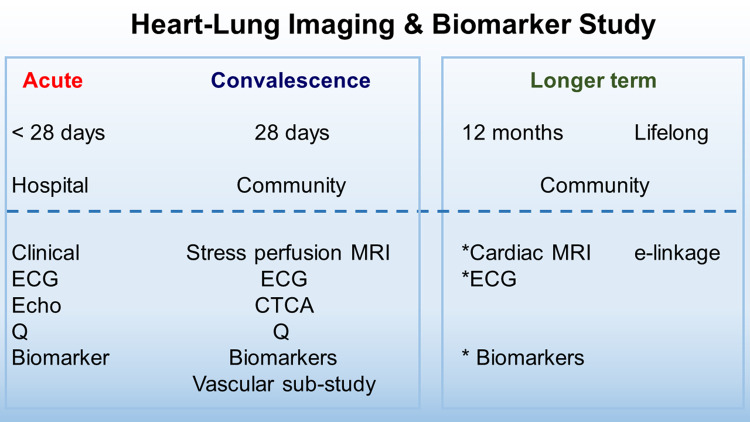
Schematic study design: flow diagram. The time periods are from hospital discharge. Abbreviations: CTCA, omputed tomography coronary angiography; ECG, electrocardiogram; Q, questionnaires; MRI, stress perfusion cardiac magnetic resonance imaging.

Multiparametric, stress perfusion, cardiovascular MRI, CT coronary angiography (CTCA), and a 12-lead ECG will be acquired at ∼28 days post-discharge. The study is designed to assess for imaging evidence of multiorgan injury (*Figure 2*).

### Setting

**Figure 3:**
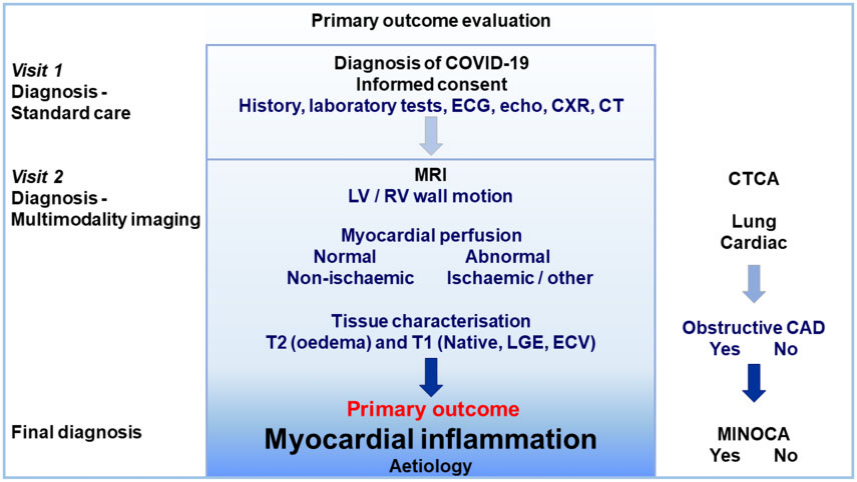
Primary outcome evaluation.

The study will involve multiple centres in the West of Scotland (population 2.2 million) including the Queen Elizabeth University Hospital, the Royal Infirmary in Glasgow, and the Royal Alexandra Hospital in Paisley. These hospitals provide secondary care services for NHS Greater Glasgow and Clyde Health Board (*Figure 3*).

### Patient identification

Patients with a diagnosis of COVID-19 will be identified from clinical databases. The clinical pathways include, but are not limited to: (i) Emergency Medicine and in-patient wards; and (ii) laboratory records.

### Eligibility criteria

The inclusion criteria are: (i) age >18 years old; (ii) history of hospital attendance or hospitalization for COVID-19, confirmed clinical diagnosis e.g. case definition according to criteria of the World Health Organisation and Health Protection Scotland), laboratory test [e.g. polymerase chain reaction (PCR)], and/or a radiological test (e.g. CT chest or chest X-ray); (iii) able to comply with study procedures; and (iv) able to provide written informed consent. The radiology results will be reported by accredited radiologists according to contemporary, national guidelines.

The exclusion criteria are: (i) contra-indication to CMR, e.g. severe claustrophobia or metallic foreign body; (ii) contra-indication to intravenous adenosine, i.e. severe asthma, long QT syndrome, second- or third-degree atrioventricular (AV) block, and sick sinus syndrome; and (iii) lack of informed consent.

### Screening

We aim to screen a near-consecutive cohort of patients diagnosed with COVID-19. A screening log will be prospectively completed. The reasons for being ineligible, including lack of inclusion criteria and/or presence of exclusion criteria, will be prospectively recorded. This information will characterize selection bias, if any.

### Recruitment

One hundred and eighty patients will be enrolled following written informed consent. If a consented patient is subsequently found to be ineligible, unless consent is withdrawn, they will remain included in the study population, including consent for long-term follow-up using linkage of electronic government and patient records (EPRs).

## Diagnosis of COVID-19

A diagnosis of COVID-19 will be based either laboratory evidence of SARS-CoV-2 infection using a PCR test on a biospecimen or a radiological diagnosis consistent with COVID-19 but biospecimen negative.^54^ The laboratory tests used are either the Roche Cobas 6800 or Seegene SARS-CoV-2 tests.

## Study procedures

The current protocol involves two visits. The first visit involves informed consent and baseline assessments. The second visit occurs at 28 days after discharge. A third visit is intended at 1 year, contingent on additional funding and a protocol amendment being secured. A proposed vascular biology substudy involves a gluteal skin biopsy after 28 days. The participants will be invited to consent for life-long follow-up using electronic record linkage without direct contact.

The study assessments involve gathering information from standard of care procedures and research tests. The standard of care information includes demographics, medical history (including multimorbidity), limited examination, laboratory and radiological tests, cardiology tests (including an ECG and an echocardiogram if clinically indicated), and treatment. The research assessments at both visits include blood and urine samples, a 12-lead digital ECG (Beneheart R3, Mindray), health status questionnaires, and assessments of adverse events. Visit 2 involves cardiovascular imaging, including stress perfusion MRI and CTCA, and an ECG. By designating imaging during the convalescent phase, at 28 days post-discharge, the participants are not anticipated to be infectious. This approach aligns with other contemporary studies, such as the International Severe Acute Respiratory and Emerging Infection Coronavirus Clinical Characterisation Consortium-4 (ISARIC-4C) study.^55^ Since MRI and CTCA are appropriately not performed during the acute phase, some pathologies that might have been detected acutely may have resolved by 28 days. Therefore, all clinical information obtained during Visits 1 and 2 will be used to inform the diagnosis of myocardial injury.

### Cardiovascular disease and risk factors

Prior cardiovascular disease includes diabetes, hypertension, myocardial infarction, heart failure, stroke, and transient ischaemic attack.

Cardiovascular risk factors include age, cigarette smoking, obesity, hypertension, hyperlipidaemia, and glycated haemoglobin. Cardiovascular risk will be described using the established JBS3 and Heart Age Scores.

## Outcomes

### Primary outcome

The primary outcome for our study is a diagnosis of myocarditis (myocardial inflammation). The relevant endotypes are (1) myocardial inflammation due to (1.1) myocarditis, (1.2) ischaemia, or (1.3) stress (Takotsubo) cardiomyopathy, (1.4) combination; (2) myocardial infarction; (3) indeterminate; or (4) none.

Myocardial inflammation is caused by the immune response to virus infection, autoimmune disease, ischaemic injury, or toxic agents.^49^ MRI provides a non-invasive approach to characterizing acute and chronic myocardial pathology. Expert consensus recommendations for the CMR-based diagnosis of definite myocardial inflammation include one T2-based criterion (global or regional increase of myocardial T2 relaxation time or an increased signal intensity in T2-weighted CMR images), with at least one T1-based criterion [increased myocardial T1, extracellular volume, or late gadolinium enhancement (LGE)].^49^^,^^50^ Having just one criterion may support a diagnosis of probable myocardial inflammation. A key attribute of the imaging protocol in our study involves the prioritized assessment of myocardial perfusion using pharmacological stress testing with intravenous adenosine. This approach is intended to permit classification of myocardial injury as being ischaemic or non-ischaemic, to facilitate classification of myocardial infarction vs. ischaemic or non-ischaemic myocardial inflammation. Information on myocardial perfusion will provide insights into coronary microvascular dysfunction that may, potentially, occur following COVID-19.^51^ Information from CTCA will clarify the presence or absence of obstructive CAD and MINOCA.

#### Consensus-based diagnosis of the primary outcome

A panel of three or more cardiologists will assess the clinical information to make a diagnosis (endotype) and related certainty (Not/unlikely = no; Probable/very = yes) before and after disclosure of the MRI and CTCA findings. The diagnosis will be based on consensus. This approach reflects the uncertainty in determining a diagnosis in patients with myocardial injury. The diagnosis draws upon clinical information and test results, rather than any single test modality in isolation.

### Secondary outcomes

A prioritized secondary outcome is the endotype for myocardial injury, including myocardial infarction type according to the 4th Universal Definition of MI,^47^ and myocarditis (myocardial inflammation, ischaemia, or stress cardiomyopathy).^49^^,^^50^

### Patient-reported outcome measures

We aim to assess the impact of cardiovascular complications on health status, well-being, and physical function. We will prospectively collect patient-reported outcome measures (PROMS) in order to assess for associations with the cardiovascular complications of COVID-19, reflected by the primary and secondary outcomes. Self-reported health status will be assessed using the generic EuroQOL EQ-5D-5L questionnaire and the Brief Illness Perception Questionnaire (Brief-IPQ).^56^^,^^57^ We will utilize the Patient Health Questionnaire-4 (PHQ-4) to assess for anxiety and depressive disorders.^58^ Participants will be invited to complete these questionnaires at each visit. The Duke Activity Status Index (DASI) provides a measure of functional capacity, and a higher score reflects greater physical function.^59^ The International Physical Activity Questionnaire–Short Form (IPAQ-SF) measures the types of intensity of physical activity and time spent sitting that people do as part of their daily lives. The score reflects total physical activity in MET-min/week and time spent sitting.^60^

## Biomarkers

In order to research the mechanisms of cardio-pulmonary and renal involvement of SARS-CoV-2 infection, we will measure circulating biomarkers of inflammation (CRP, ferritin, IL-6), cardiac injury (troponin I, NT-proBNP), renin–angiotensin system (aldosterone, sACE2), and haemostasis [coagulation screen, Clauss fibrinogen, D-dimer, FVIII (one stage), VWF antigen and VWF:GP1ba, antithrombin, protein C, and free protein S)], and renal function (albumin:creatinine ratio) and their changes over time.

**Table 1:** Secondary outcomes

Secondary outcomes	
**Health status**	Illness perception (Brief IPQ) Anxiety/depression (PHQ4) EQ-5D-5L The Duke Activity Status Index (DASI) The International Physical Activity Questionnaire – Short Form (IPAQ-SF) Serious adverse events (SAEs)
**Cardiac MRI**	Impaired LV systolic function Increased LV end-diastolic volume Myocardial oedema (T2 map) Myocardial inflammation (native T1 map) Late gadolinium enhancement Increased extracellular volume Impaired myocardial perfusion Impaired RV systolic function Increased RV end-diastolic volume
**CT chest and angiography**	Consolidation Pulmonary artery diameter Thrombus, main pulmonary artery Microthrombii
**Renal**	Urine albumin:creatinine ratio
**Biomarkers**	Circulating biomarkers of inflammation (CRP, ferritin), cardiac injury (troponin I, NT-proBNP), vascular injury (aldosterone, sACE2, IL-6) and haemostasis [coagulation screen, Clauss fibrinogen, D-dimer, FVIII (one stage), VWF antigen and VWF:GP1ba, antithrombin, protein C, and free protein S)], and renal function (albumin:creatinine ratio)
**Feasibility**	Participant withdrawal rate
**Exploratory outcomes**	Vascular biology changes Computational modelling of co-registered imaging to develop novel biomarkers ECG abnormalities Renal MRI—kidney size, oedema/inflammation, apparent diffusion coefficient (ADC), a quantitative parameter calculated from diffusion-weighted imaging (DWI)

The measurements will be undertaken in a central laboratory, blind to the other clinical data. The associations between the circulating concentrations of these mechanistic biomarkers, including their changes over time, and the primary and secondary outcomes will be assessed.

## Cardio-pulmonary and renal MRI

Cardiovascular MRI is the reference diagnostic method for myocardial injury, including myocarditis and acute cardiomyopathy. Stress perfusion MRI using intravenous adenosine enables dynamic imaging of myocardial blood flow during stress and rest conditions. In-line pixel mapping enables fully quantitative read-out of myocardial blood flow (mL/min/g tissue), classified at a subsegmental level (32 myocardial segments) with the percentage extent of myocardium with impaired perfusion during stress (% ischaemic burden).^61–66^ All of this information can be spatially mapped with LV function, tissue characteristics revealed by T2-mapping, native T-mapping, LGE, and extracellular volume (ECV).^52^ In this study, the modified Lake Louise criteria will be used to diagnose definite myocardial inflammation [T2 map + T1 (native T1, LGE, or ECV abnormal)] or probable myocardial inflammation (either T2 or T1 abnormal).^49^^,^^50^ In order to limit selection bias, renal dysfunction is not an exclusion criterion. Patients with severe renal dysfunction [glomerular filtration rate (GFR) <30 mL/kg/m^2^] will be considered for contrast MRI according to local radiology protocols.

SARS-CoV-2 causes vascular dysfunction and microthrombotic angiopathy.^10^ Our scientific hypothesis is that microvascular dysfunction underlies the cardiovascular toxicity caused by this infection through primary and secondary mechanisms. Direct virus infection of injured endothelial cells, disrupting the integrity of the endothelial barrier, permits virus infection of deep tissues, causing myocarditis and nephritis. Myocarditis and ischaemia may induce cardiomyopathy leading to left and right ventricular dysfunction. Secondary consequences of COVID-19 including hypoxia, inflammation, and vasospasm impaired tissue perfusion, leading to myocardial ischaemia.

MRI will be undertaken at 3.0 Tesla (Siemens PRISMA) ∼28 days after discharge. This timepoint may be considered as the subacute, convalescent phase. The rationale for undertaking the multiparametric, stress perfusion cardiovascular MRI at this timepoint is to determine whether there is persisting evidence of myocardial injury and/or MI, LV function, and incidental pathology (e.g. mural thrombus) in the convalescent phase. The patients will be advised to withhold cardiovascular medications and to avoid caffeine-containing drinks for 24 h before the scan.

MRI will also provide information for incidental findings in the chest (e.g. pulmonary arterial thrombus) and abdomen. Imaging of renal anatomy, size, and tissue characterization will be exploratory.

## Computed tomography

The CT scanner has 320 detectors enabling full heart coverage within a single heartbeat (Aquilion ONE, Canon). Intravenous metoprolol will be used where required to control the heart rate (target 60 b.p.m.) and sublingual glyceryl trinitrate will be given to all patients immediately before the scan acquisition. An initial low radiation dose helical scan of the thorax will be acquired for comprehensive assessment of the lungs. A contrast bolus timing scan will be acquired which will provide information on cardiopulmonary transit times. Non-contrast and contrast-enhanced angiographic breath-hold ECG-gated volumes will be acquired timed for optimum pulmonary and systemic arterial (coronary) opacification.

CTCA will provide information on the presence and extent of coronary calcification (calcium score), CAD, and whether CAD is obstructive (flow-limiting) including the CAD-RADS score. Intracardiac thrombus will be assessed. Late enhancement ECG-gated CT will be acquired to assess for delayed enhancement (scar)^67^ and ECV calculation.^68^ Pulmonary vascular imaging will assess pulmonary vascular thrombosis (embolism) including CT obstruction score, cardiopulmonary transit times, and measures of raised pulmonary artery (PA) and right heart pressures [PA, caval and azygous dimensions plus hepatic inferior vena cava (IVC) reflux]. CT will also characterize pulmonary features associated with COVID infection (percentage ground-glass opacity, percentage consolidation, and CO-RADS score) plus pre-existing lung damage (percentage chronic obstructive pulmonary disease). CT will also assess for signs of osteoporosis and sarcopenia as frailty markers.

The CT and MRI findings will be correlated with the other clinical data. Cardiac and extracardiac findings will be reported and managed according to local standards of care. Patients with severe renal dysfunction thought to be at risk of acute kidney injury as determined by local radiology clinical protocols will undergo non-contrast CT.

## Natural history

In order to assess the natural history, longer term follow-up for health outcomes will be undertaken using electronic record linkage, removing the need for participants to undergo further research visits after the 12-month visit.

## Exploratory analyses

The scans will be pseudoanonymized, i.e. identifiers removed and assigned a unique study number to enable linkage. The scans will be shared with research collaborators for cardiovascular modelling to better understand the relationships between tissue pathology, blood flow, and function.

COVID-19 infection and treatment are associated with changes on the ECG. The changes include alterations in heart rate, conduction, and ventricular repolarization. Drug treatment for COVID-19 may prolong the QTc interval. Whether these changes persist after the acute phase and their relationships to myocardial pathology are unknown. Paper and/or digital ECGs will be acquired, de-identified, and provided to the University of Glasgow Electrocardiography Core Laboratory for automated analysis. The ECG measures will be linked with the clinical and imaging data.

We will undertake exploratory research into the vascular biology of COVID-19 infection. This work will be undertaken in collaboration with cardiovascular scientists and virologists in the University of Glasgow. We will study the vascular biology of cells and molecules (e.g. RNA and cytokines) implicated in SARS-CoV-2. Informed consent will be obtained for post-mortem examination in the event of death during the study period. Histology samples of the heart will be examined for features of myocarditis, MI, and microvascular disease. The histopathological findings will be linked to the CMR findings.

## Feasibility

This study has been developed with input from members of a multidisciplinary research team and the Scientific Strategy Group of the University of Glasgow. The study has been peer reviewed by panel members of the Chief Scientist Office of the Scottish Government. The study has been reviewed by the Patient and Public Involvement group of NHS Greater Glasgow and Clyde Health Board.

## Statistical considerations

The statistical analyses will be pre-defined according to a Statistical Analysis Plan.

### Sample size calculation

The primary outcome is myocarditis (myocardial inflammation). To detect an association between a history of pre-existing cardiovascular disease and the incidence of myocardial inflammation (myocarditis) at 2–4 weeks, we have assumed 25% of patients with prior cardiovascular disease and the incidence of myocardial inflammation in those with/without prior cardiac problems to be 33% and 10%, respectively. To have 80% power to detect this difference will require 140 participants (35 with cardiac problems, 105 without) to be scanned. We aim for 160 patients to attend the imaging visit, anticipating that 10–15% of the participants may have incomplete imaging data due to technical reasons such as imaging artefact or claustrophobia.

Pre-specified subgroup analyses are intended for patients without cardiovascular disease, as defined by the absence of (i) prior cardiovascular disease and (ii) obstructive CAD on CTCA. Given the public health significance of COVID-19, interim reports may be undertaken.

### Blinding

Outcome assessments (endpoint adjudication) will be undertaken in blinded fashion. The primary outcome evaluation (myocardial injury endotype) will be adjudicated by a panel of cardiologists blind to the clinical status of the patient and performed according to a pre-specified charter.

## Trial management and timelines

The trial will be conducted in line with the current *Guidelines for Good Clinical Practice in Clinical Trials* and STROBE guidelines.^69^ The Study Management Group (SMG) includes those individuals responsible for the day-to-day management of the study including the chief investigator, project manager, and representatives from the sponsor. The role of this group will be to facilitate the progress of the study, ensure that the protocol is adhered to, and take appropriate action to safeguard participants and the quality of the study itself. Decisions about continuation or termination of the study or substantial amendments to the protocol will be the responsibility of the sponsor. A scientific steering group will oversee the study. This study is designed to be undertaken and reported rapidly in response to the global need for information about COVID-19.

## Ethics

The CISCO-19 study is approved by the UK National Research Ethics Service (Reference 20/NS/0066).

## Sources of funding

CISCO-19 is an investigator-initiated clinical study that is funded by the Chief Scientist Office of the Scottish Government (COV/GLA/Portfolio project number 311300). The funder has no role in the study design, conduct (non-voting TSC member), data analysis and interpretation, manuscript writing, or dissemination of the results. C.B, C.D., N.S., R.M.T. are supported by the British Heart Foundation (BHF RE/18/6134217).

The MRI study involves imaging and analyses technologies provided by Siemens Healthcare and the National Institutes of Health

The trial is co-sponsored by NHS Greater Glasgow & Clyde and the University of Glasgow.

## Registration

The ClinicalTrials.gov identifier is NCT04403607.

## Discussion

Our observational, multimodality, imaging cohort study will prospectively gather information on the cardiovascular complications and their clinical significance in COVID-19. A relatively unselected approach to patient enrolment will minimize selection bias beyond those who do not survive or who are unable to comply with the protocol. The findings will be generalizable to patients in the convalescent phase of the illness and informative for the natural history. On the other hand, the findings will not necessarily be generalizable to all patients with COVID-19, since our enrolment strategy focuses on survivors following the acute phase of the illness.

Cardiovascular MRI will be used to clarify clinical endotypes according to contemporary guidelines.^49^^,^^50^ Quantitative measurements of myocardial blood flow at stress and rest will enable focused research into coronary microvascular dysfunction that may be secondary to endotheliitis caused by SARS-CoV-2. CT imaging will clarify the presence and relevance of CAD and MINOCA, lung pathology, and pulmonary arterial thrombosis. Renal MRI will be undertaken on an exploratory basis to assess kidney size (differential volume) plus both global values and cortico:medullary ratios for T1, T2, and the apparent diffusion coefficient (ADC) for correlation with renal function. Renal dysfunction is not an exclusion criterion, reflecting the open approach to enrolment. Taken together with a comprehensive clinical assessment, laboratory tests (including renal function and urine albumin:creatine ratio), and circulating biomarkers, our study will characterize multisystem involvement of SARS-CoV-2 infection.

High-sensitivity troponin is a cardiac protein that is ubiquitously released from injured cardiomyocytes. However, troponin is not cause specific, and circulating concentrations may increase due to hypoxia, hypotension, and renal failure, as well from direct cardiac toxicity. It is unclear whether cardiovascular involvement in COVID-19 is mainly secondary to severe pneumonia, or whether there is direct viral infection of the heart and blood vessels. A recent expert consensus guideline highlighted the pivotal value of CMR in the diagnosis of myocardial inflammation due to viruses, autoimmune disease, ischaemic injury, and toxic agents. MRI is diagnostically useful to identify endotypes for stratified therapy. We will assess whether this could be the case in COVID-19.

To our knowledge, CISCO-19 is the first to apply stress perfusion CMR to assess and quantify abnormalities in myocardial perfusion that may be secondary to microvascular dysfunction. The information on myocardial perfusion will help classify patients with myocardial injury into ischaemic or non-ischaemic groups. Stress perfusion MRI is not usually undertaken in patients with myocarditis, and quantitative measurements of myocardial blood flow are a key attribute of our study design. We aim to advance new knowledge into the pathogenesis of myocardial inflammation (ischaemic vs. non-ischaemic) and into the associations between the aetiology of disease and abnormalities in myocardial perfusion. Recent advances in fully quantitative, in-line pixel mapping of myocardial perfusion are uniquely enabling the quantification of myocardial blood flow in near real-time without the need for time-intensive, off-line post-processing. In addition, multimodality imaging involves multiparametric cardiovascular MRI and CTCA during the same visit. As such, our study will provide methodologically robust estimates of persisting myocardial injury and the impact on the physical and mental well-being of the participants. The results should be helpful to inform clinical management strategies for the diagnosis and management of patients recovering from COVID-19. Our study will provide complementary information to add to the growing body of knowledge on multisystem involvement in COVID-19.

We will collect information on participants’ characteristics at baseline including demographics, anthropometry, cardiovascular and medical history, and health status. The participants will be invited to give informed consent for life-long follow-up by linkage of EPRs. Using multivariable analyses, we will link the patients’ characteristics at baseline to observations during longer term follow-up in order to characterize the natural history of this condition.

Our imaging research will clarify the prevalence and clinical significance of cardiopulmonary injury (notably myocardial inflammation) in patients with COVID-19, which is a major knowledge gap in the NHS. By adopting an all-comers approach, we will identify patients with myocardial inflammation that is subclinical (i.e. not diagnosed) or clinically overt. By correlating the MRI findings with troponin and other measures of cardiovascular injury, such as BNP, our results will potentially inform NHS care pathways to use these blood tests in a more directed manner for the clinical management of patients with COVID-19. Currently, there are no disease-modifying therapies for myocarditis, including due to SARS-CoV-2.

Overall, our study will add new knowledge on the natural history of COVID-19 in a comparatively unselected population. Our study will create a unique biorepository of clinical samples and images, which in turn may be exploited by scientists undertaking mechanistic research into the vascular biology of SARS-CoV-2 infection, and cardiovascular modelling.
